# Development and Testing of the Kids Hurt App, a Web-Based, Pain Self-Report App for First Nations Youths: Mixed Methods Study

**DOI:** 10.2196/48370

**Published:** 2025-03-03

**Authors:** Karlee Francis, Julie Francis, Margot Latimer, Hayley Gould, Shante Blackmore, Emily MacLeod

**Affiliations:** 1 Eskasoni Health Centre, Eskasoni, Nova Scotia, Canada; 2 Dalhousie University, Halifax, Nova Scotia, Canada; 3 Tajikeimɨk Mi’kmaw Health & Wellness, Membertou, Nova Scotia, Canada; 4 School of Nursing, Dalhousie University, 6299 South Street, Halifax, Nova Scotia, B3H 4R2, Canada, 1 9022293844; 5 IWK Health, Halifax, Nova Scotia, Canada; 6 Nova Scotia Health, Sydney, Nova Scotia, Canada; 7 Nova Scotia Health, Halifax, Nova Scotia, Canada

**Keywords:** app, eHealth, pain, Indigenous, First Nations, children, youths, mobile phone

## Abstract

**Background:**

First Nations children and youths may have unique ways to convey their health needs that have not been recognized by health providers. This may contribute to the disparity between high rates of mental health and physical pain and low rates of treatment for the conditions they experience. Evidence suggests that a colonial history has resulted in poor experiences with the health care system, lack of trust with health providers, and miscommunication between clinicians and patients. Contemporary ways, using both Indigenous and Western knowledge, are needed to bridge the gap in communicating pain.

**Objective:**

The aim of this qualitative study was to test the usability and clinical feasibility of the Kids Hurt App with First Nations youths and clinicians working with youths.

**Methods:**

Using a Two-Eyed Seeing approach, the Kids Hurt App was developed using concepts from validated mood and pain assessment apps combined with community-based research that gathered First Nations youths and clinicians perspectives on quality, intensity, and location of pain and hurt. The Kids Hurt App contains 16 screens accessible on any web-based device.

**Results:**

In total, 3 rounds of low-fidelity testing (n=19), 2 rounds of high-fidelity testing (n=20), and 2 rounds of clinical feasibility testing (n=10) were conducted with First Nations youths (10‐19 years) to determine the relevance, validity, and usability of the Kids Hurt App. High-fidelity testing was also conducted with 15 clinicians after completing the high-fidelity youth sessions. Youths had constructive suggestions that were used to improve the app in subsequent rounds of version testing. There was one main discrepancy between youths and clinicians related to preference for how best to visually convey pain. The youth’s preference was maintained in the app.

**Conclusions:**

All youths in all rounds of testing indicated that they would use the Kids Hurt App if it was available to them in a health care setting, with most clinicians noting that the app would be useful in practice.

## Introduction

### Overview

Indigenous peoples in Canada are comprised of 3 distinct groups: First Nations, Inuit, and Métis. They reside across Canada, from large cities to small isolated communities [1]. In comparison to non-Indigenous peoples, Indigenous peoples are the fastest-growing cohort in Canada with a significantly younger population [1]. Indigenous children younger than 14 years of age represent 25.4% of the total Indigenous population, while the same age group in the non-Indigenous population only compromises 16% [1]. Repeated reports of racism in health care, such as those in the In Plain Sight report describing Joyce Echaquan’s fatal health care experience in Quebec [2], combined with research findings [3,4] suggest that health care providers need to understand the structural and historical factors that contribute to the current racial disparities faced by Indigenous peoples [5]. A legacy of colonialism and present-day racism is a determinant of health that is unique to Indigenous peoples so efforts need to be made to create better understanding, appropriate assessment, and improved care for health conditions such as pain [6].

While 1 in every 5 Canadian people experiences chronic pain, this number is significantly higher in Indigenous communities, and most types of pain are more prevalent in Indigenous peoples [7]. Indigenous children are particularly vulnerable to experiencing pain. In one study examining pain diagnoses, a large sample of First Nations children (n=2631) who were compared to an age-, sex-, and location-matched sample of non–First Nations children, demonstrated that the First Nations cohort experienced significantly more physical pain–related diagnoses for 10 of the 13 pain indicators studied [7]. Further to this, while ear- and throat-related diagnoses were the 2 most diagnosed pain conditions for both groups, the First Nations cohort was significantly less likely than the non–First Nations cohort to visit a specialist for these same conditions, demonstrating inequities in diagnosis and treatment [7]. Additionally, these data showed that children (0‐9 years) who had a physical pain diagnosis were more likely to have a mental health diagnosis in adolescence, a finding only evident in the non–First Nation cohort who were seemingly able to access mental health care [7].

Cultural variances in pain expression have also been found between Indigenous and non-Indigenous children and youths [8,9]. Latimer et al [8] concluded that Mi’kmaq children were stoic and hid their pain. Children reported that they would not cry when enduring pain and preferred instead to be “brave” or “tough it out” [8]. These findings led researchers to conclude that health clinicians may not have ways to accurately assess or document the level of pain a child or youth is experiencing. Similar findings of stoicism were reported in Indigenous peoples in Australia, with participants often being described as quiet about their pain and not reporting pain due to higher pain tolerance, fear of Western medicine, or intercultural communication difficulties [10]. Clinicians are trained to use numerical or face pain scales to assess pain; however, only minimal research has been done to determine if these self-report pain scales are culturally appropriate for use with Indigenous children, and indeed to our knowledge, none in First Nations children [11,12]. Community members reported that numerical pain rating assessment scales are confusing, and it is difficult to attach hurt or pain to one measure such as a face or number [9]. The evidence suggests that there is a clinical practice gap in assisting Indigenous youths to convey their pain to health providers. This study endeavored to find a culturally appropriate mechanism to support youths in sharing their pain and to address reports of a lack of clinician’s ability to recognize pain, which creates a barrier to appropriate health care [13]. In response to these needs, the Kids Hurt App [14] was cocreated with Indigenous and clinical partners. It was created with the notion that Indigenous peoples have many effective ways of communicating knowledge that include visual images and storytelling. This led the developers to explore how both Indigenous and Western ways could be used to develop a better assessment tool that puts the technology in the hands of the youths. The aim of this qualitative study was to test the usability and feasibility of the Kids Hurt App with First Nations youths and clinicians working with youths.

### Background

#### Two-Eyed Seeing

The project used a Two-Eyed Seeing (TES) approach, a term coined by Elders Murdena and Albert Marshall, which recognizes the benefit of seeing Indigenous knowledge and experience from one eye while also seeing strengths from another eye’s perspective that is different from one’s own [15]. The Western perspective is most typically how health care is currently delivered. When both eyes are used, the ultimate benefit is achieved by effectively enhancing the health of Indigenous peoples through the practical sharing of knowledge [16].

#### Digital Health Interventions for Indigenous Children and Youths

Although there is a growing body of literature on digital health and app-based interventions to manage pain in children and youths, research on the development and usability of app-based interventions for Indigenous peoples is limited. In a review of digital health solutions for Indigenous mental well-being across Canada, the United States, Australia, and New Zealand, Hensel et al [17] indicated that co-designed app-based interventions had been shown to reduce anxiety and depression symptoms and contribute to well-being. The Aboriginal and Islander Mental Health Initiative for Youth App, or AIMhi-Y, was developed in consultation with Indigenous youths from Australia and the Torres Strait Islands [18]. The youths identified apps as a potential way to mitigate barriers to accessing help and highlighted the need for a strength-based approach [18,19]. A randomized controlled trial found that iBobbly, a suicide prevention app developed in consultation with Indigenous youths living in Australia, had only a minimal, nonsignificant impact on psychological well-being outcomes, although qualitative data indicated the app was helpful and that construct validity issues may have been the reason why there was not a significant impact [20]. To date, there is no research on the development or usability of apps for Indigenous children and youths in Canada [21].

#### Development of the Kids Hurt App

The Kids Hurt App was developed by the Aboriginal Children’s Hurt and Healing Initiative for children aged 10‐19 years. Funding for the app development and testing was received from the Canadian Institutes of Health Research (FRN# 162455 & SCA-145102), Indigenous Health Nursing Research Chair, Dalhousie University (FRN# 167603), and IWK Health Foundation. The Aboriginal Children’s Hurt and Healing Initiative is a broad partnership, consisting of Indigenous community leaders, clinicians, elders, youths, researchers from universities, and health delivery systems. In keeping with Ownership, Control, Access and Possession (OCAP) principles [22], the app is owned by communities involved in this research study. OCAP is a registered trademark of the First Nations Information Governance Centre and describes the principles of ownership, control, access, and possession to ensure that First Nations have control over data processes including ownership and how their information is used. The app was developed to facilitate pain communication between clinicians and Indigenous youths in a culturally safe manner. It is not meant to replace the history-taking step of a health care interaction but rather serves to augment the quality of communication exchange by giving youths an opportunity to share their pain story in a comfortable, culturally safe way.

The app was developed using TES to ensure both Indigenous and Western concepts and content were included. In total, 16 “screens” or interfaces were developed. Multimedia Appendix 1 provides an example of the final 16 screens developed. Previous versions of these screens were used throughout various stages of testing.

The first app screen asks users their gender and age (see Screen 1 in Multimedia Appendix 1), with the subsequent screen providing them with the option to create an avatar or choose a predesigned one (see Screen 2-3 in Multimedia Appendix 1). First Nations youths were involved in the development of the avatar designs through initial content validity testing to ensure that visual representation was present. Once an avatar is chosen, it is prompted to answer a series of responses to physical and emotional pain prompts. The inclusion of both physical and emotional pain prompts provides an example of how TES [15] was used throughout the development of the app. Earlier design ideas for the app had only physical pain prompts; however, community feedback suggested that incorporating both physical and emotional pain prompts would better represent the Indigenous holistic view of health, which involves “a healthy balance of 4 elements or aspects of wellness: physical, emotional, mental, and spiritual” [23]. While this version of the Kids Hurt App does not incorporate all 4 wellness aspects, emotional and physical pain were chosen because they were the most reported types of pain in previous research [7]. Future app iterations are expected to include spiritual and mental dimensions. There is also evidence that physical and emotional pain are interconnected, and the app presents an opportunity to highlight this for both youths and clinicians.

**Figure 1.**
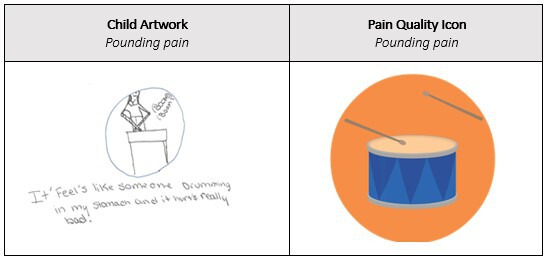
Pain quality icon development.

Additional incorporation of TES [15] involved the use of the International Association for the Study of Pain assessment tool guidelines [24] in the development, ensuring three key assessment features were embedded into the app: (1) pain location (see Screen 4-6 in Multimedia Appendix 1), (2) pain quality (see Screen 7 in Multimedia Appendix 1, and (3) pain intensity (see Screen 8 in Multimedia Appendix 1). Pain quality icons were conceptualized based on previous art-based research with Indigenous youths [8,9] as well as the Iconic Pain Assessment Tool [25] and the Pain Quality Icon (QuILT) [26] as illustrated in Figure 1.

Pain intensity was initially captured using 2 scales: a 10-point “face scale,” mimicking the Faces Pain Scale—Revised (FPS-R) [27], and a newly developed “Jar of Hurt,” building on the concept of the “Pieces of Hurt” tool [28] and respecting the nation’s language and word for “pain” which is “hurt” [8]. A jar was used as a common, everyday item that would provide the imagery of filling something up with pain or hurt. In addition, details of *where* users were when they got hurt (ie, school) and *what* they were doing (ie, playing) were also included with options based on common places of injury noted in the First Nations Regional Health Survey [29].The inclusion of the *where* and *what* aspects of the app allowed the incorporation of storytelling (see Screen 9-10 in Multimedia Appendix 1), an approach with significant cultural ties for Indigenous populations [30].

**Figure 2.**
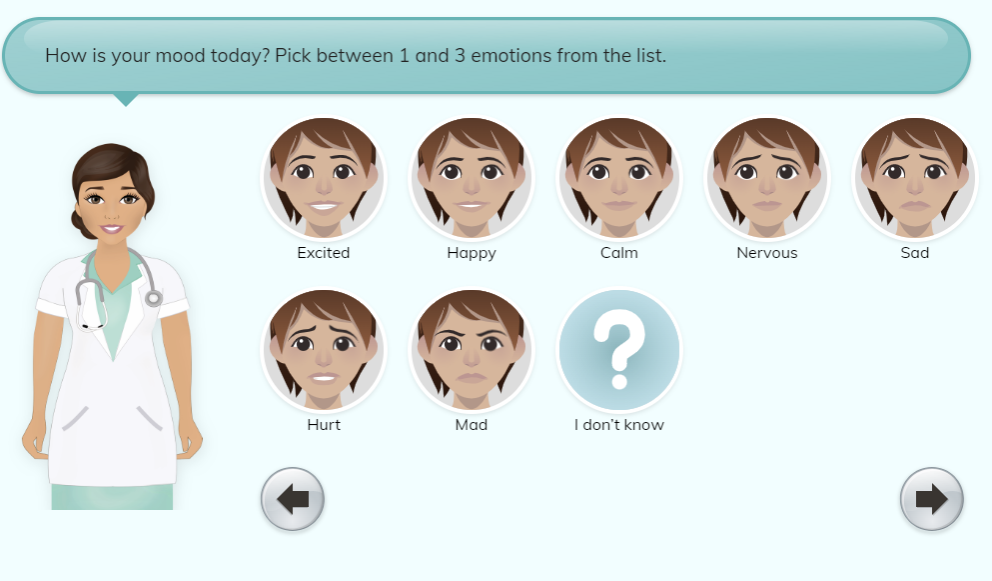
Emotional pain icons.

After completing questions regarding the user’s physical pain, users are then prompted to provide information on their emotional pain through a series of similar screens (see Screen 11-14 in Multimedia Appendix 1). Emotional pain icons were initially chosen based on 22 common emotions identified from 20 validated mood apps (Multimedia Appendix 2). Through the initial content validity testing with First Nations youths, these 22 emotions were narrowed down to 8, which were identified as most likely to be used to describe their emotional mood. The resulting 8 emotions, both “negative” and “positive,” provided a range of options and avoided bias in responses (Figure 2). Emotional pain questions focused on users’ current emotional state (happy, sad, and anxious), intensity of those emotions (using the 10-point Jar of Hurt), and any physical symptoms that may be associated with their emotional pain (ie, chest tightness).

Upon answering both the physical and emotional pain questions, information is presented on a final storyboard screen (see Screen 15 in Multimedia Appendix 1).

## Methods

### Study Design

Once an initial version of the app was finalized three types of testing took place: (1) low-fidelity testing, (2) high-fidelity testing, and (3) clinical feasibility testing. This testing or development strategy mirrored the process published by Stinson et al [31].

### Ethical Considerations

Prior to project initiation, the study was approved by both an Indigenous and tertiary pediatric hospital research ethics board (Mi’kmaw Ethics Watch & IWK Health Research Ethics Board; #1017831). Informed consent was obtained from all participants or their parents or guardians prior to taking part in study procedures. Youth participants received a CAD $10 (US $6.98) gift card after completing the testing. Clinician participants were not compensated. Data were deidentified immediately following data collection sessions.

### Participants

First Nation youth participants were recruited through social media platforms and community organizations or contacts to complete low-fidelity, high-fidelity, and clinical feasibility testing. Clinicians recruited from a First Nation Health Centre and a pediatric health center unit specializing in emotional or physical pain–related services (rheumatology, pain clinic, etc) also completed high-fidelity testing. Eligibility criteria for youth participants included (1) self-identifying as Indigenous, (2) residing in a First Nation community, and (3) being between 10 and 19 years of age.

### Data Collection

#### Overview

The study took place over a 5-year period between April 2017 and March 2022. Significant delays were experienced due to the COVID-19 pandemic. All youth data collection sessions were completed within the youth’s Indigenous community to ensure that local support resources were available if a youth felt distressed. A trained mental health clinician was available as a resource for each session, and smudging was offered for youths who took part in the clinical feasibility sessions.

#### Low-Fidelity Testing

Low-fidelity testing is a qualitative usability approach where youths were able to view a paper-based version of the app. Youths completed a demographic survey prior to reviewing the app screens. In total, 3 rounds of low-fidelity testing took place. During each round, youths were shown paper-based screenshots of the Kids Hurt App and asked what they liked and disliked about the design through a series of semistructured questions. Sessions were audio-recorded and transcribed for clarity and accuracy. After each round of testing, feedback was reviewed and integrated into the app. Screenshots of the updated version of the app were then shown to the next round of participating youths until no further changes were suggested based on the methods of Stinson et al [31].

#### High-Fidelity Testing

High-fidelity testing is similar to low-fidelity testing; however, participants were able to review a fully functioning version of the app rather than paper-based screenshots. Youths completed a demographic survey prior to using the app, and all sessions were audio-recorded and transcribed. Two rounds of high-fidelity testing were completed with participating youths to determine functionality and acceptability and to allow for any suggested revisions to be shared. The app was viewed on an iPad, and the youths worked through the app in the presence of the research assistant. At the start of each session, youths were asked to complete the app by themselves and to talk out loud about any likes, dislikes, or difficulties. The length of time it took for youths to complete the app from start to finish was recorded. Then, they were asked to go through the app for the second time with the research assistant who asked them semistructured questions. Once finished, they were asked open-ended questions on their overall thoughts regarding likes, dislikes, ease of use, and whether they would use the app in a health care setting if it was available. Feedback from each round was incorporated until no further changes were suggested (2 rounds). Interviews with the youths were audio-recorded in each of the 2 rounds to maintain the integrity and accuracy of the findings.

After the youths completed the high-fidelity testing, clinicians were then invited to take part in high-fidelity testing. Clinicians were invited to use the app and then answer a series of questions either through a digital survey or during a focus group. Focus group sessions were transcribed to ensure data accuracy. Questions assessed the clarity and importance of the various app components and also allowed space for participants to provide suggestions on what they would like to change or add for each screen. Demographic questions were also asked to determine participant’s profession and types of experience.

#### Clinical Feasibility Testing

Clinical feasibility testing was completed with youths to help determine if the app works well, if it is easy to use, and if youths found it relevant for use in their care. Youths were asked to work through the app with the research assistant present and then answer a series of questions in a digital survey format. Questions ranged from demographic questions to those regarding the app’s usefulness as well as functionality. Once an initial 5 youths had completed their review of the app, changes were incorporated, and an additional 5 youths were invited to take part. After 2 rounds of clinical feasibility testing with very positive results and minimal changes, it was determined that a third round was not needed.

### Data Analysis

Demographic survey responses from all rounds of testing were analyzed using the SPSS Statistics (version 24; IBM Corp) database system to provide an overall breakdown of age and gender. Field notes and transcribed interviews were reviewed to determine any changes needed for subsequent rounds of testing. Revisions associated with more large-scale app functionality that were unable to be incorporated between testing rounds, such as incorporating a feature to track pain over time, were added to a database for future recommendations.

## Results

### Low-Fidelity Testing

**Figure 3.**
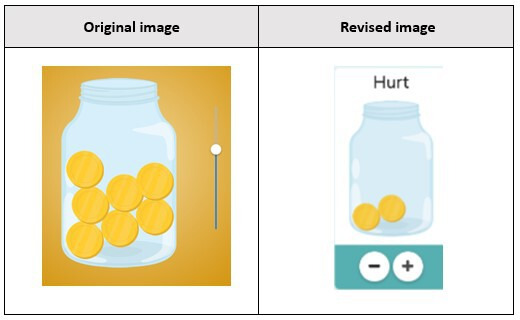
Jar of Hurt—plus or minus button addition.

A total of 19 youths participated in 3 rounds of low-fidelity testing (round 1: 7 youths, round 2: 6 youths, and round 3: 6 youths). In total, 63% (n=12) of the participants were identified as female. The average age for all participants was 13 (SD 2.46) years. In the first round of low-fidelity testing, 1 participant suggested that the Jar of Hurt scale should use a plus or minus button to increase or decrease the amount of hurt rather than the original “slider” option (Figure 3). In subsequent rounds, youths were shown both the slider and the plus or minus option and asked their preference. The majority of youths (n=11) preferred the plus or minus option. The directions for describing how to use the Jar of Hurt were also identified during low-fidelity testing as needing clarification. A total of 6 of the 19 youths identified this issue. “I don’t know, it’s a little confusing. It sounds complicated” (Female, 14 years). The language was simplified, and it was agreed that it would be further reviewed in the high-fidelity rounds.

**Figure 4.**
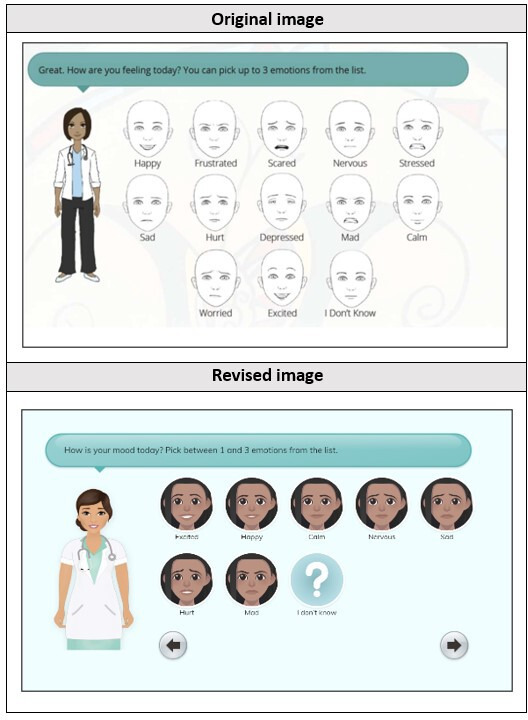
Emotion faces—revised. Original image depicts 10 emotions rather than the finalized 8 emotions, as this was an initial mock-up screenshot for testing purposes only.

The final noted change from low-fidelity testing was an overall dislike of the way the emotion face icons looked. One youth described the faces as “creepy.” To address this, the facial icons were redesigned to look like the user’s avatar rather than a generic face as illustrated in Figure 4.

### High-Fidelity Testing

A total of 20 youths participated in the 2 rounds of high-fidelity testing (round 1: 10 youths and round 2: 10 youths). In total, 70% (n=14) of participants were female, and the average age for youth participants was 16 (SD 2.46) years. The average time it took youths to complete the app was 3.61 minutes (216.6 seconds; SD 48). All youth participants identified enjoying the app and agreed it would be helpful to them.

It was pretty helpful. I know when you go to your doctor, you don’t really want to open up. Sometimes you’re nervous. At least the app helps explain how you feel.

[Female, 17 years]

Confusion remained regarding the use of the Jar of Hurt with 2 of 10 youths in the first round of testing describing it as confusing. As a result, examples of an empty jar and a full jar were added beside the plus or minus buttons to provide a visual of how to increase or decrease the amount of pain or hurt (see Screen 7 and 8 in Multimedia Appendix 1). After this change was implemented, no confusion was noted in the remaining high-fidelity testing round.

Participants also suggested revisions to the “I took something harmful” icon in the “how you were hurt” section. Initially, this icon included a skull and crossbones to symbolize poison; however, youths suggested that something more representative of drugs or alcohol should be included.

I think this would be something chemical, based on the picture and skull thing. But I think something that can be added is maybe drugs or alcohol. Because the person may not always feel comfortable saying it but hitting it on the button without thinking and if it’s right in front of you, they may be honest.

[Female, 18 years]

**Figure 5.**
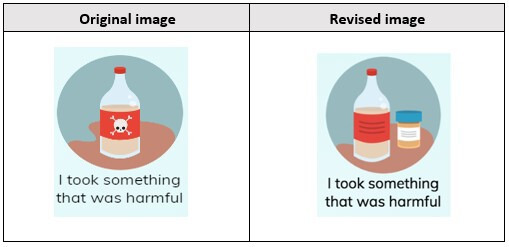
“Harmful” icon changes.

This icon was revised to better reflect youth participant’s perception of which icons are most relevant to them (Figure 5).

**Figure 6.**
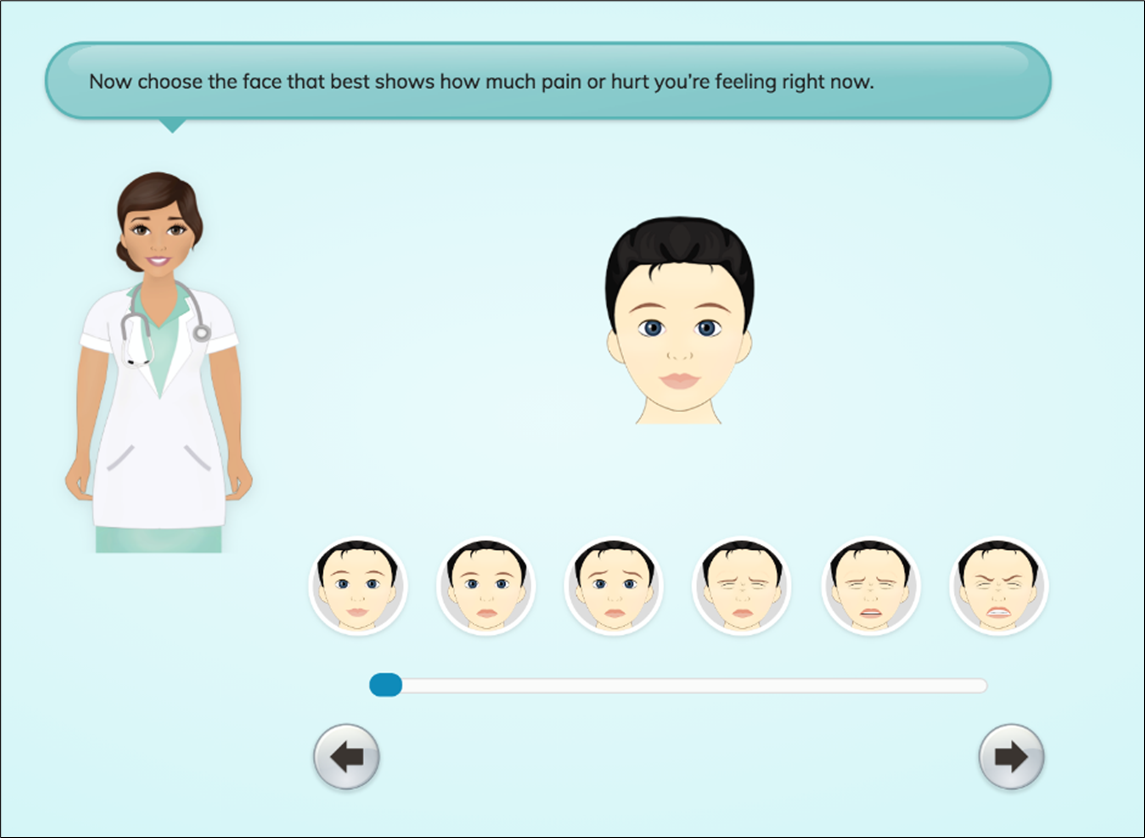
Faces Pain Scale—Revised expressions avatar adapted faces scale.

**Figure 7.**
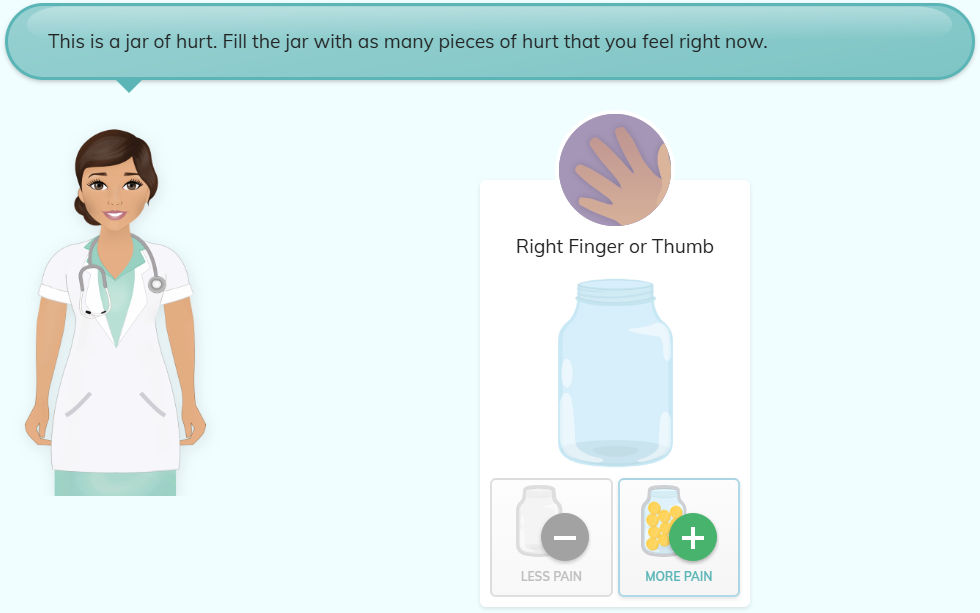
Jar of Hurt scale.

A final revision resulting from the youth high-fidelity sessions was determining a preference for which scale to use to represent the quantity of pain. Youths were shown a scale with facial images similar to those in the FPS-R and superimposed in the avatar [27] (Figure 6) and the Jar of Hurt scale (Figure 7) and asked which they felt would best allow them to quantify their pain. The majority (n=12, 60%) preferred the Jar of Hurt scale. One youth shared their reason why:

If you had to choose one option, I would say the jar scale would be a lot better. Because maybe some people, they know how to hide it. Their pain. So, I’d say the jar scale is a lot better.

[Male, 16 years]

In addition to youths, a total of 15 clinicians participated in high-fidelity testing. In total, 10 clinicians completed this through a digital survey, and 5 participants took part in a focus group session. Most survey respondents were nurses (n=7, 70%), and focus group participants included physicians, physiotherapists, nurses, and psychologists.

In total, 14 of 15 clinicians found the app to be moderately or extremely useful. Like youths, clinicians were asked to identify their preference between the Jar of Hurt or the FPS-R adapted scale (Figures6  7) through the survey questions. A total of 7 of the 9 clinician respondents identified a preference for the FPS-R adapted scale.

On 2 different screens, clinicians suggested adding an “other” option. For the quality of pain screen, 4 of 5 clinicians from the focus group felt that an “other” option should be included to allow users to add their own quality of pain descriptors. Similarly, for the emotional pain screen, all clinician participants suggested adding an “other” option to allow users the flexibility of adding any emotion they felt, even if not in the presented list. These additions were made in subsequent versions of the app.

### Clinical Feasibility Testing

A total of 10 youths completed clinical feasibility testing (round 1: 5 and round 2: 5). Results from the clinical feasibility testing were very positive, with 80% (n=8) of respondents identifying that they found the app easy to use and 90% (n=9) saying they would use the app in a hospital or health care setting. No technical errors were found. Participants suggested that the Jar of Hurt method was more geared toward younger youths but also noted that they were happy to use the scale either way. Suggestions for change were mainly larger design changes identified as “future changes” including having a text or email function to send results to themselves or their care providers, having the ability to track changes in their pain or hurt over time, and including translation in their Indigenous language.

## Discussion

### Principal Findings

In this study of clinicians and First Nations youths, 3 types of testing occurred in the development of the Kids Hurt App: low-fidelity, high-fidelity, and clinical feasibility. For the 2 groups of participants, each subsequent round of testing reduced the app modifications. Overall changes were minor, but important observations were thought to increase the usefulness of the app for users.

Recent literature has identified that app-based interventions may remove barriers to accessing care for youths [18,32,33]. Apps are accessible and may mitigate confidential concerns and concerns about stigma. Smartphone-based apps may be particularly useful, as authors from one study found that smartphones are a mainstream technology and are an advantage within the health care system [34]. While existing research suggests that Western pain assessment tools are not appropriate for Indigenous peoples [10], this study demonstrates how an app can be developed collaboratively and engage youths to share their pain respecting both Indigenous and Western knowledge systems. All youths stated that they would use the Kids Hurt App if it was available in the health care setting. Youths reaffirmed that they often found it difficult to articulate their feelings and felt that the app could help them communicate their needs. This app allows them to share their pain and help facilitate enhanced communication.

One notable finding in this study was the process of determining which pain scale to use, the “Jar of Hurt” scale or the “face scale.” In the initial development stages of the app, the “Jar of Hurt” concept was chosen based on previous research regarding the “Pieces of Hurt” tool [28]. While this research was completed with younger children than our target population, the concept of using objects rather than faces was appealing based on previous research indicating that Indigenous children are often stoic in their expression of pain and may hide their pain rather than displaying it outwardly through facial expressions [8]. Through high-fidelity testing, 60% (n=12) of youths preferred the “Jar of Hurt” scale over the “face scale,” and while it was noted in clinical feasibility testing that the scale was more geared toward younger youths, participants identified being happy to use the tool, regardless of that. Clinician preference was strongly noted for the face scale likely because of its similarity to the FPS-R scale, a well-known pain measurement tool [27]. However, given that the app was developed to provide youths with a valid way to convey their pain and hurt and to develop an alternative to Western pain assessment tools, the “Jar of Hurt” was maintained as the preference for conveying pain intensity. This finding highlights the importance of taking the time to validate patient indicators of pain consistent with different age groups and populations.

### Improved Health Care Experience

During a typical patient visit, a clinician obtains the patient’s history, current pain experience, and future needs in a short amount of time [35]. This can be challenging if a patient finds it difficult to articulate their pain and hurt for a range of reasons, such as stoicism or power dynamic, further causing delay in timely and appropriate care [34,36]. In this study, participants identified the app as “easy to use” and were able to complete the app screens in an average of 3.61 minutes (216.6 seconds; SD 48). The youths’ suggestions for app improvement, such as the Jar of Hurt being a better way to capture pain instead of a face that may hide pain, provide some indication that the Kids Hurt App may facilitate better communication, and consequently health care experience, by allowing Indigenous youths to convey their pain through an app. Other comments such as the youths who indicated pushing a button might be easier than talking could imply that the app offers a nonthreatening and safe way for youths to share their stories. Research has shown that youths have high technological literacy and are comfortable using eHealth apps [37,38]. In a generation where technology is fluent, the Kids Hurt App can give youths a voice in the health care system, in a medium they are comfortable with, to personally advocate for their physical and emotional pain care.

### Limitations

At the time of testing, the app was not yet available in the youth participant nation’s language. The current version of the app [14] is fully translated into Mi’kmaw. Communication between the provider and patient should always be clear and effective, and having health information accessible in one’s primary language is important [39]. Thus, we recommend that the app be available in the nation’s primary language in any further testing. An additional limitation is that testing was only completed with 2 First Nation communities so results cannot be assumed to be accurate for all Indigenous nations.

### Conclusions

Culturally safe care acknowledges the attitudes, knowledge, and behaviors of another’s culture in a respectful manner. If a provider encounters an Indigenous patient, it would be important to understand the patient’s unique historical legacies, ways of communicating, and any potential intergenerational trauma that may influence pain report [40]. Implications of creating and using an app that has been codeveloped by the end users (First Nations youths) increase the usefulness of the app and provide a space for the provider and patient relationship to be optimized for best care outcomes. The short app completion time is an indication of the minimal effort it takes to offer a culturally appropriate alternative to pain self-report and may reduce miscommunication and repeat visits for the same condition. Future research will include testing the app with non-Indigenous youths as well as in various health settings such as mental health, tertiary, primary care, and emergency departments. Further development of the app related to youth’s self-report of effective strategies that reduce pain and hurt is planned. The Kids Hurt App is a culturally safe and evidence-based communication tool that may allow First Nations youths to comfortably share their pain and hurt stories. Each nation may be different in its communication and will need to review the app for usability and feasibility in its own communities.

## Supplementary material

10.2196/48370 Multimedia Appendix 1 App screens.

10.2196/48370 Multimedia Appendix 2 Mood app inventory.

## Abbreviations

FPS-R: Faces Pain Scale—Revised
OCAP: Ownership, Control, Access and Possession
TES: Two-Eyed Seeing
