# Supplementary material for: Development and Testing of the Kids Hurt App, a Web-Based, Pain Self-Report App for First Nations Youths: Mixed Methods Study
Source: JMIR Hum Factors. 2025 Mar 3;12:e48370. doi: 10.2196/48370 (PMC11892538; doi:10.2196/48370)

Screen 1: Age/Gender

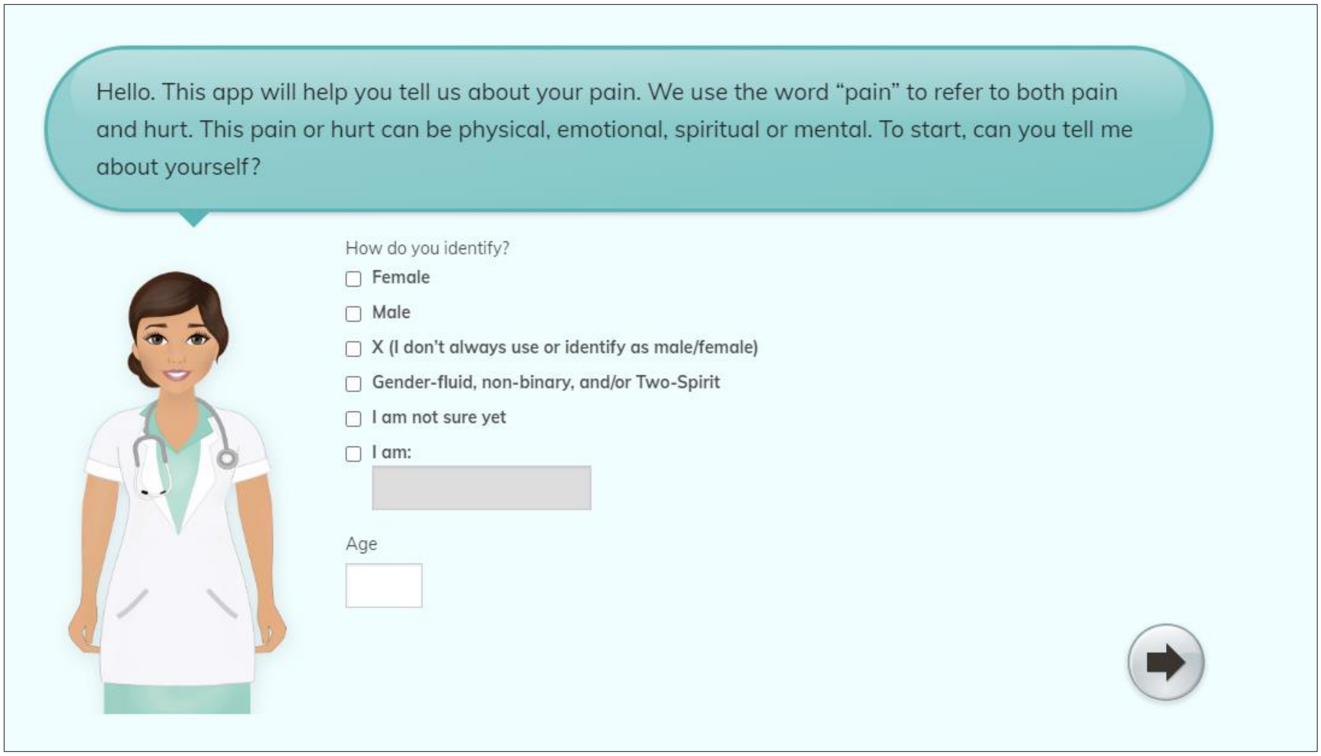

Screen 2-3: Avatar Development

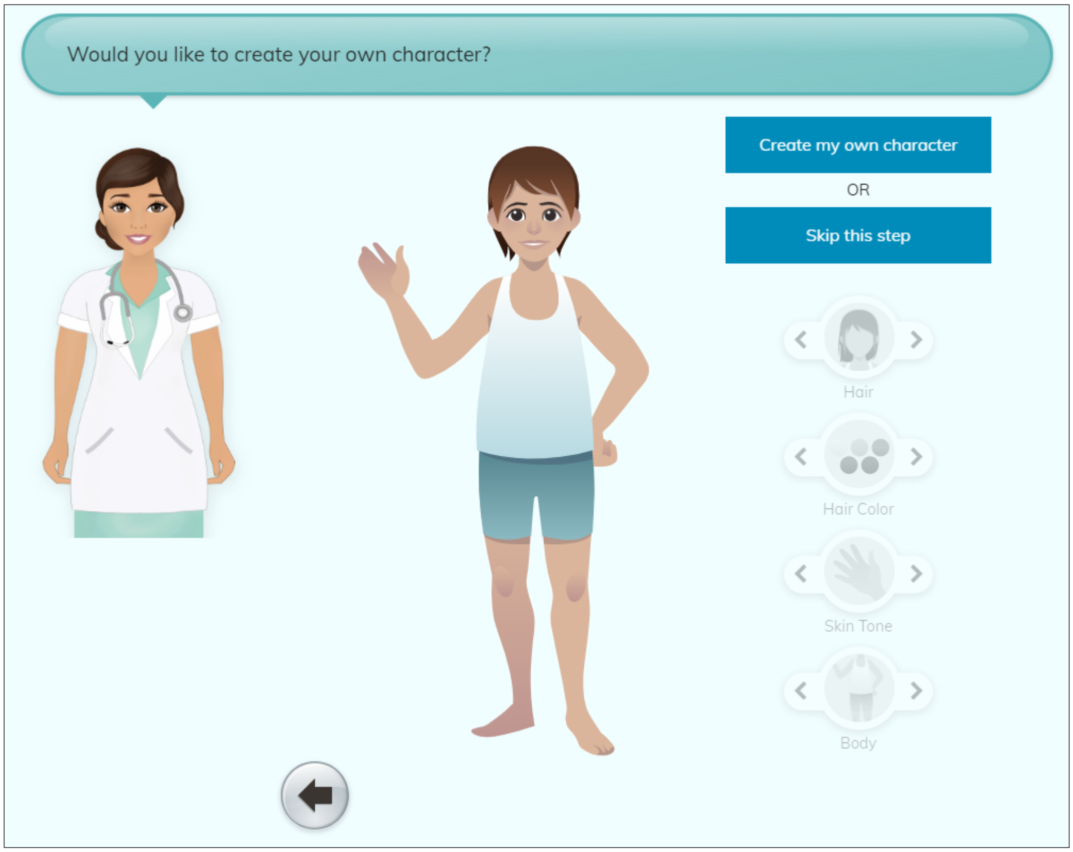

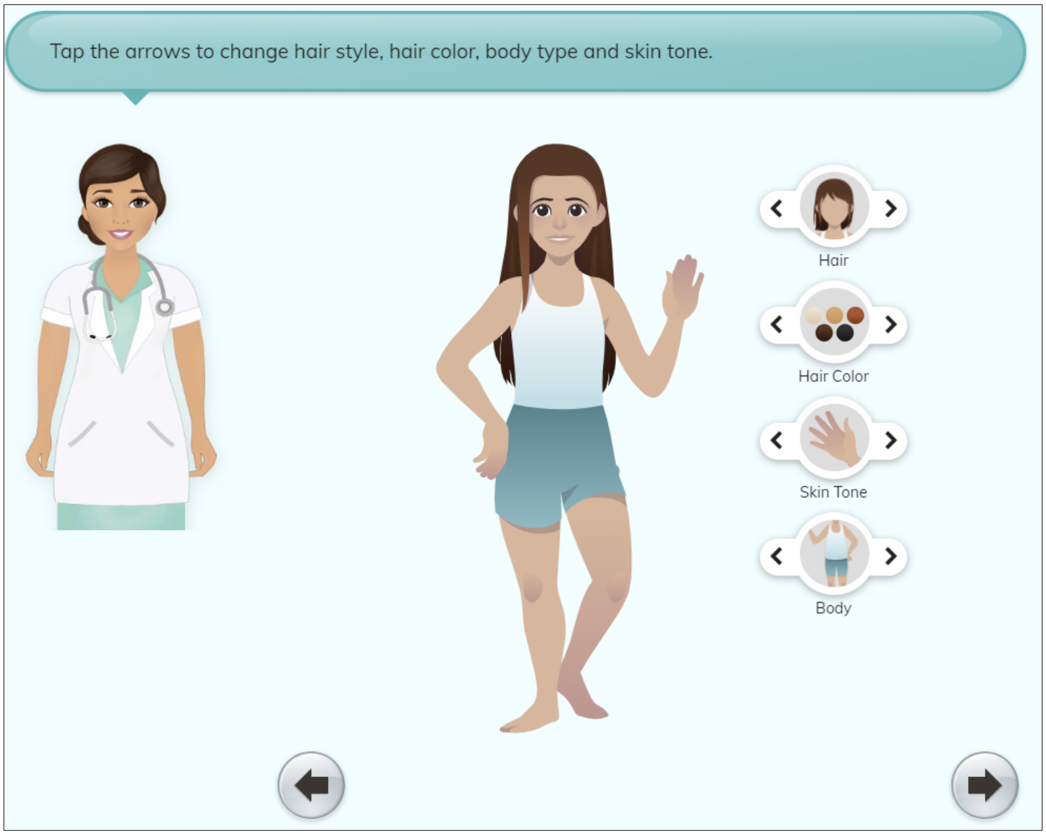

Screen 4-6: Pain Location

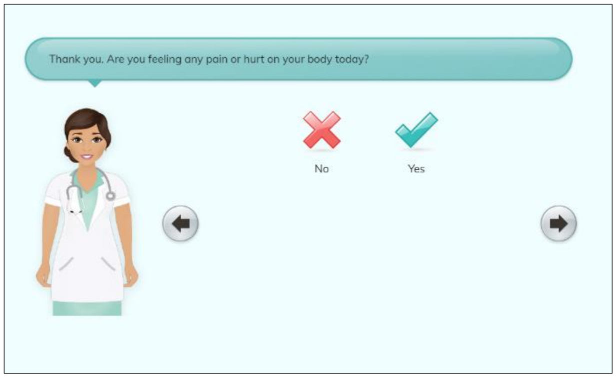

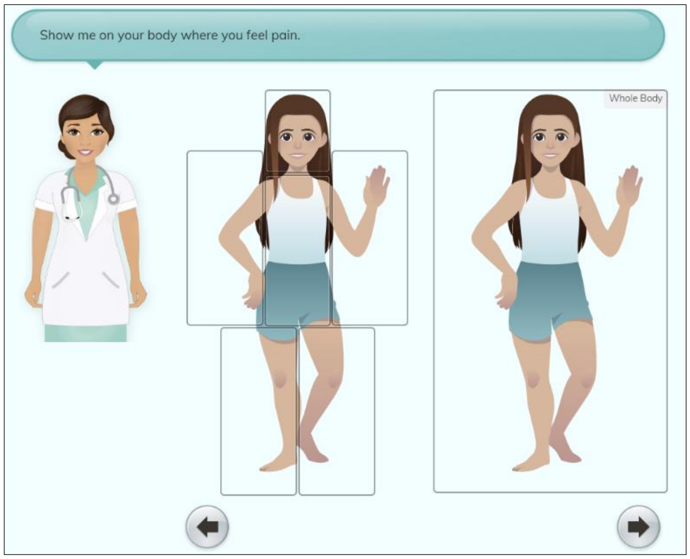

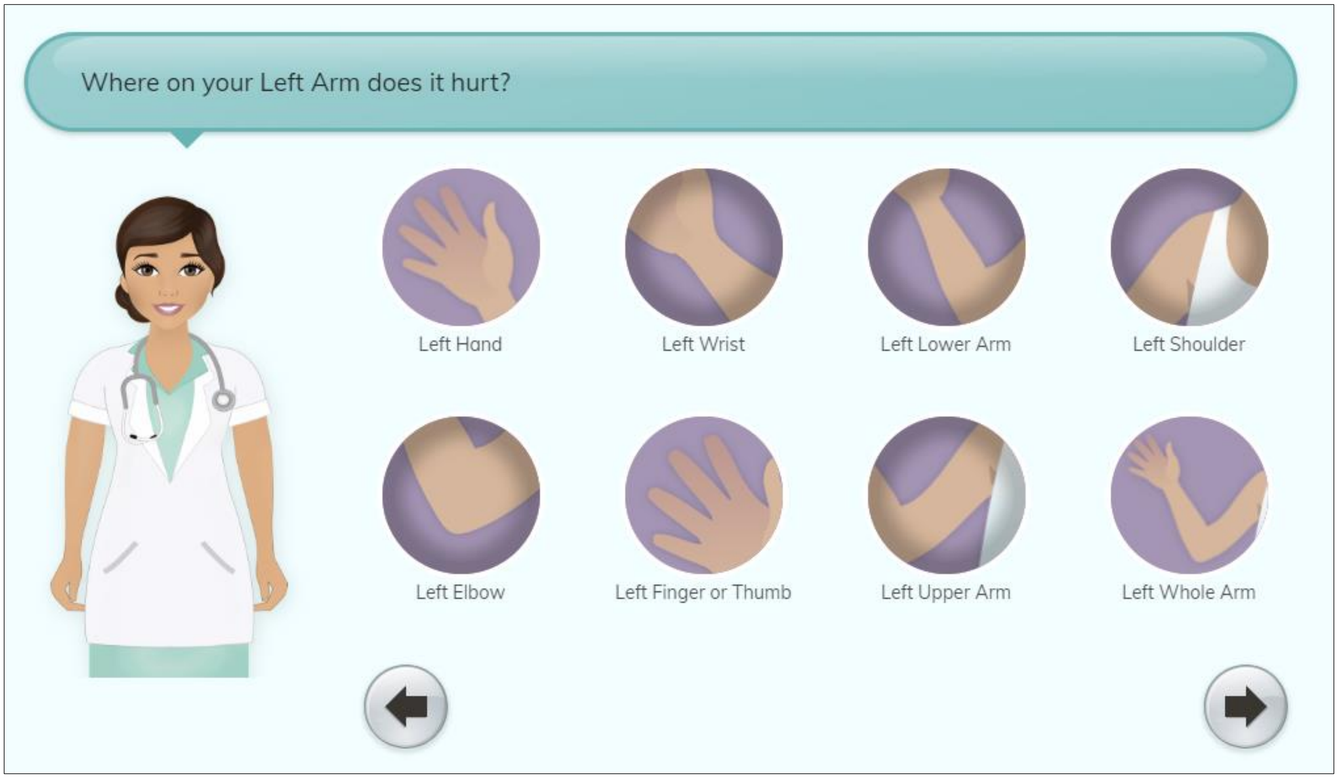

Screen 7 - Pain Quality

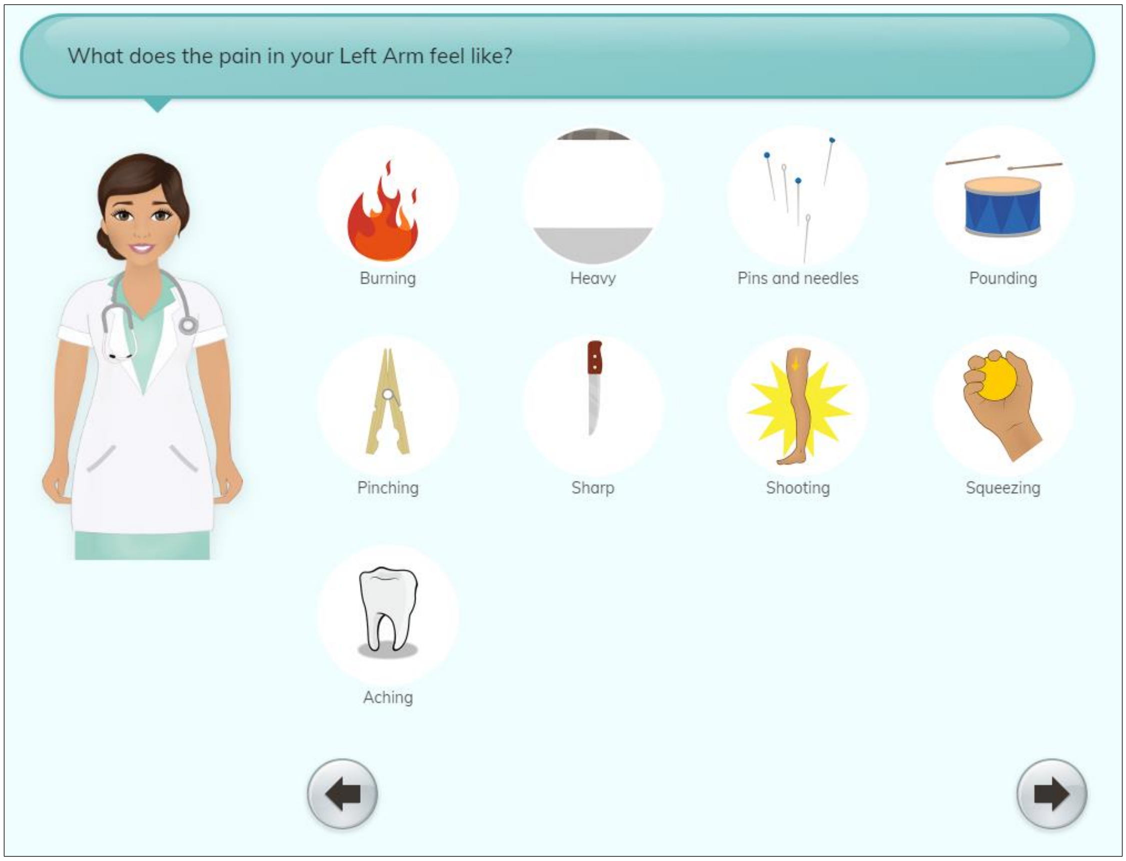

Screen 8 – Pain Intensity

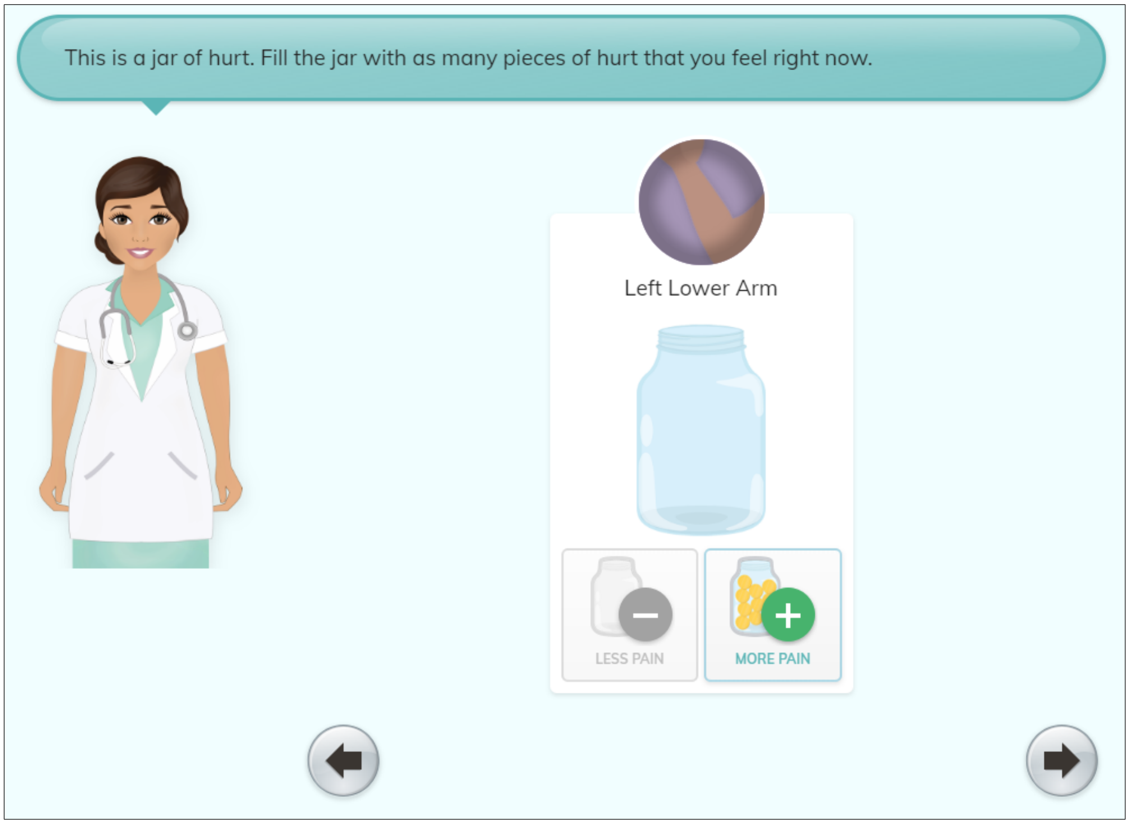

Screen 9-10 – Storytelling (where/how did you get hurt?)

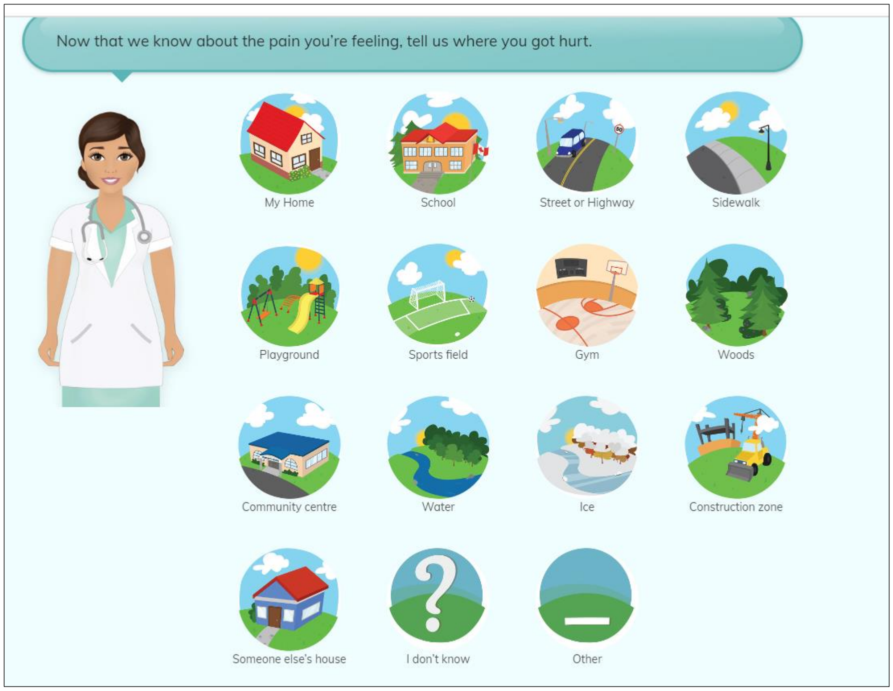

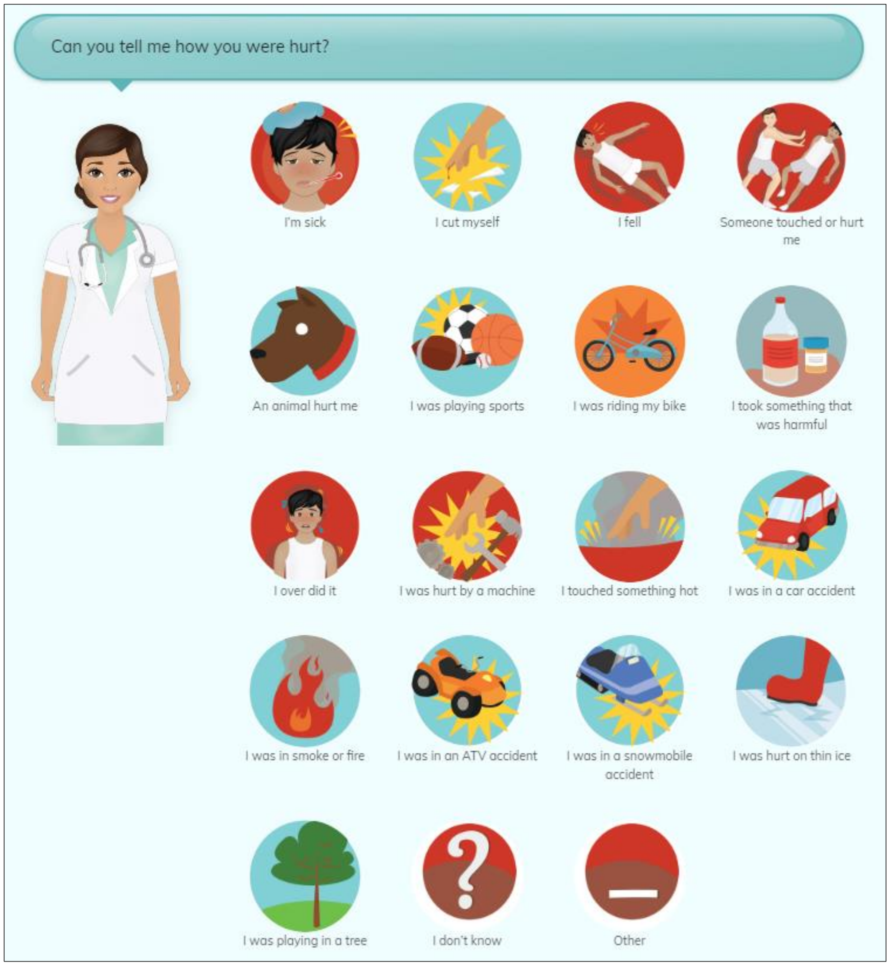

Screen 11-12 – Emotional pain

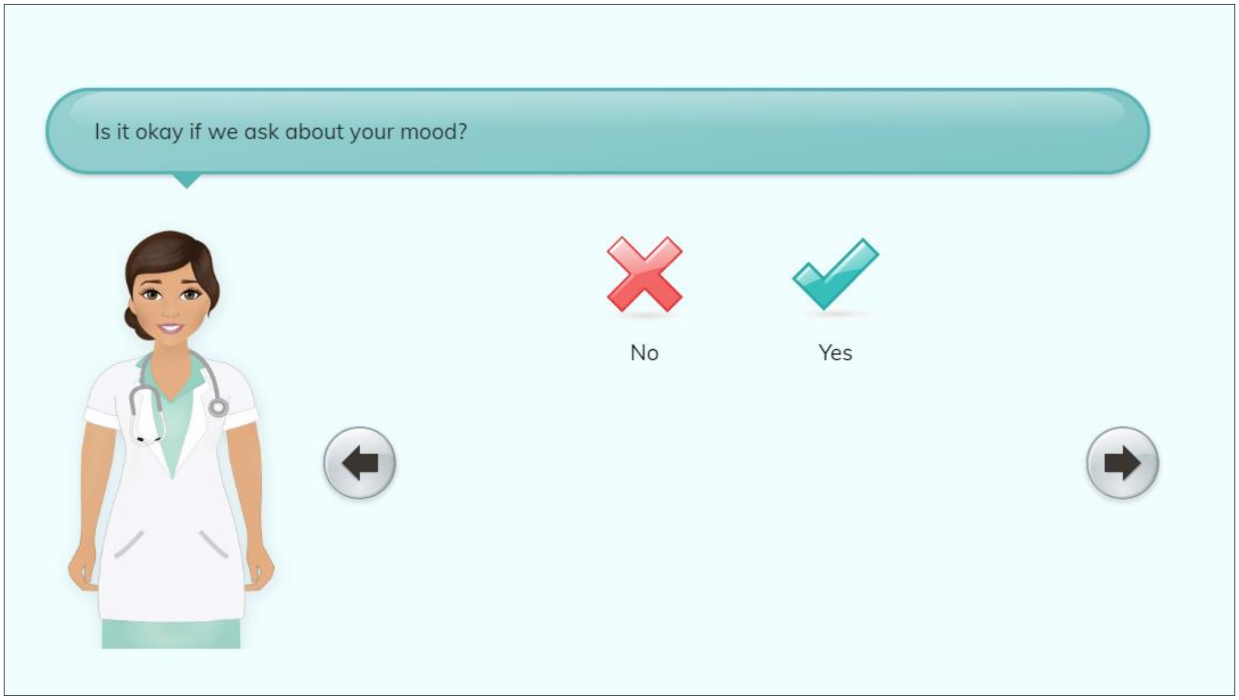

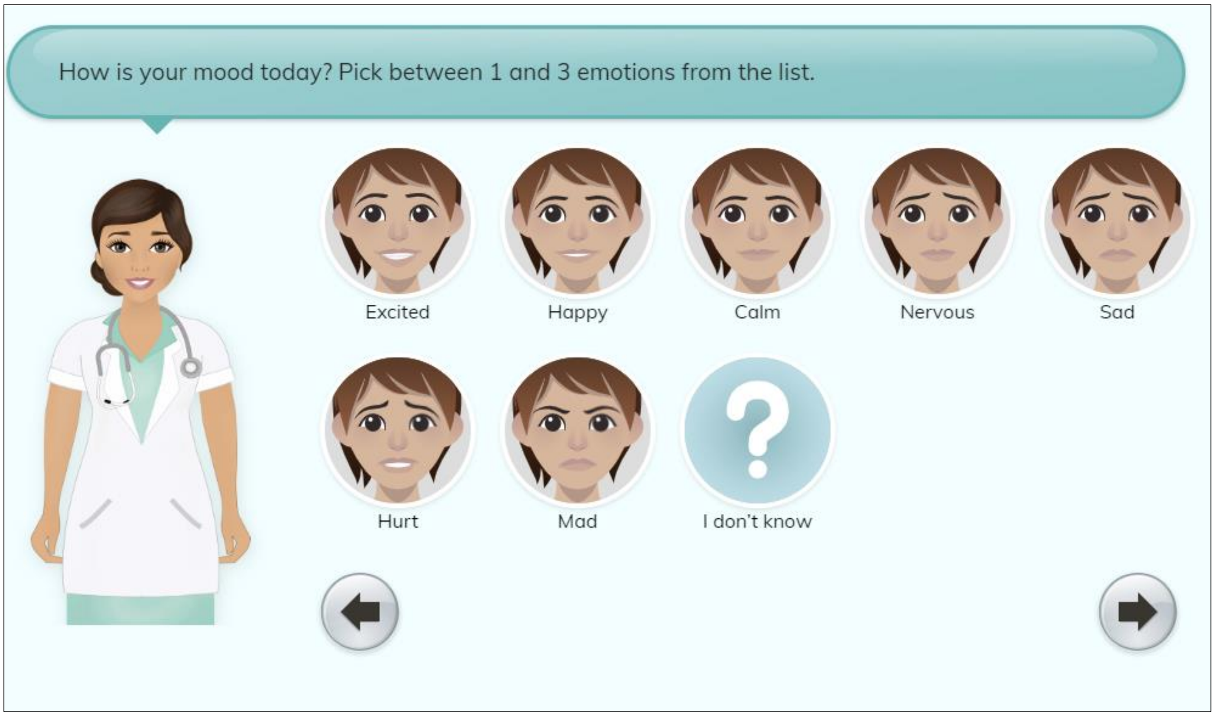

Screen 13 – Emotion Intensity

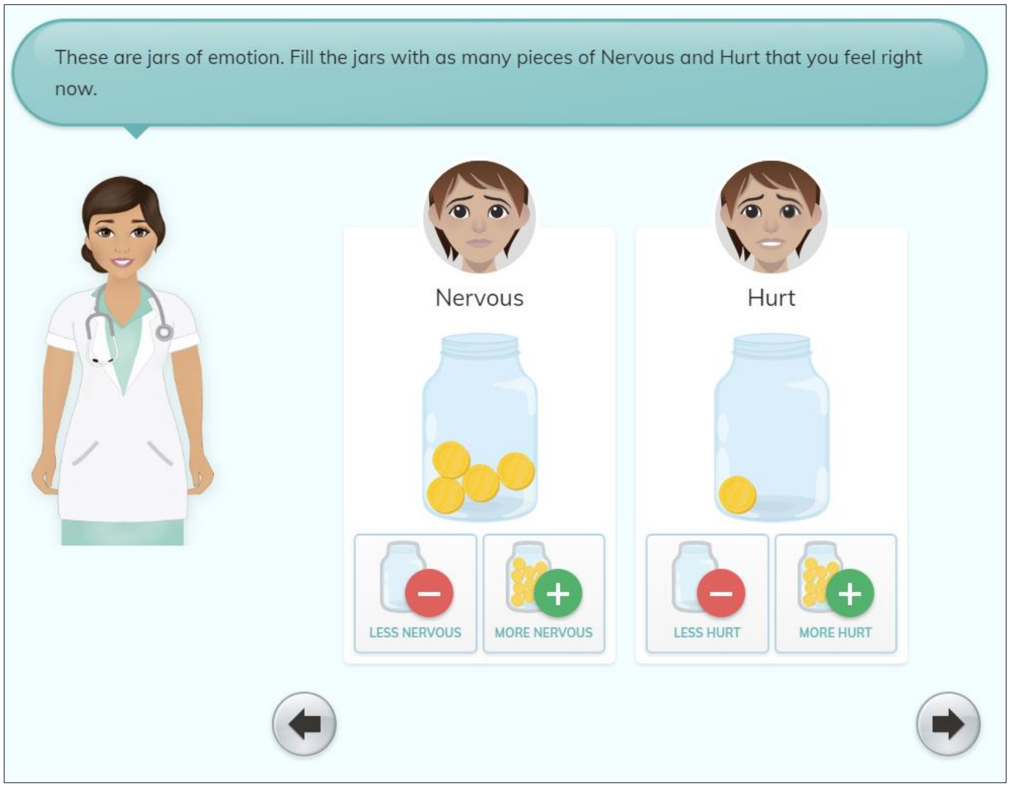

Screen 14 – Physical symptoms of emotion/mood

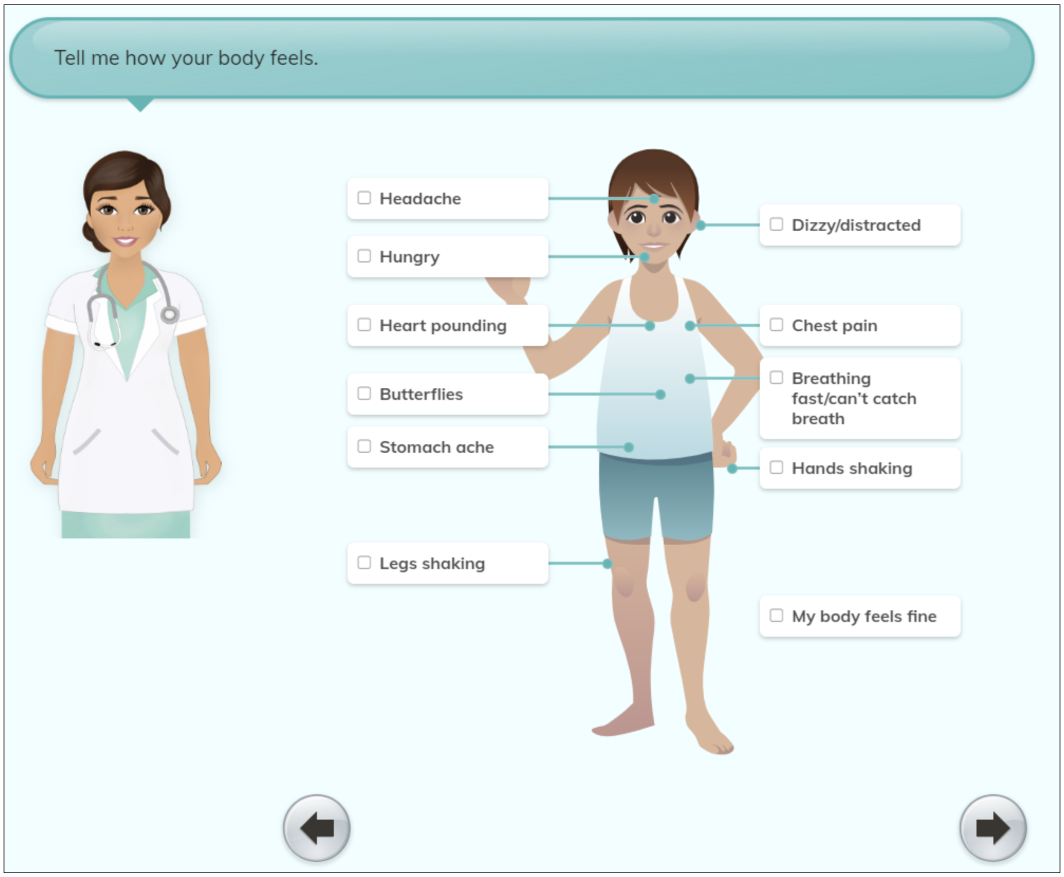

Screen 15 – Final Storyboard

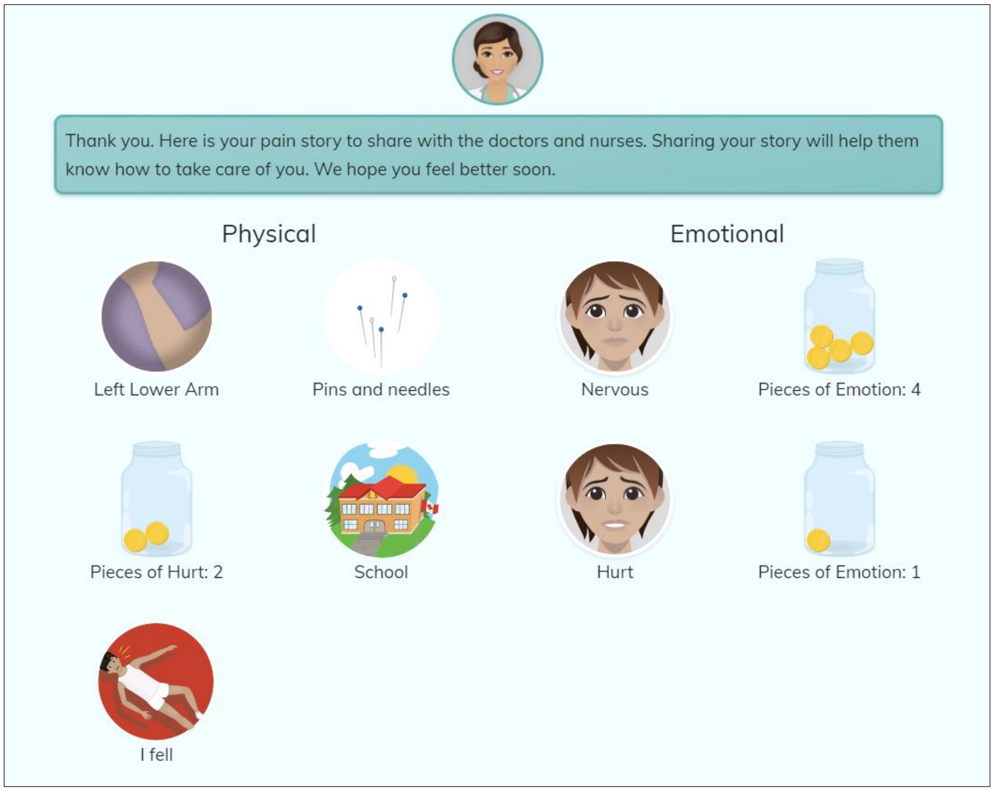

Supplement: Multimedia Appendix 1 [file humanfactors-v12-e48370-s001.pdf]
