# Supplementary material for: Development and Testing of the Kids Hurt App, a Web-Based, Pain Self-Report App for First Nations Youths: Mixed Methods Study
Source: JMIR Hum Factors. 2025 Mar 3;12:e48370. doi: 10.2196/48370 (PMC11892538; doi:10.2196/48370)
Supplement: Multimedia Appendix 2 [file humanfactors-v12-e48370-s002.pdf]

#	Screening/Assessment Tool	Acronom	Author	Date	Purpose/Objective	Target Age/Population	Domains Assessed (Primary & Secondary)	# of Items	Approx. Length	Notes on Validation	Response Format
	The Ideal Assessment			*2000+*		*5 to 18 years*		*<20*	*5 - 10 minutes*	*Validated*	*Check/uncheck, 0-3 items, yes/no*
**1**	Beck Youth Inventories	BYI	Beck, JS., Beck, AT., Jolly, JB., & Steer, RA.	2001, 2005	To provide a broad assessment of a child or adolescent's current mental health. The individual inventories may also be used in isolation to assess specific aspects of child and youth mental health.	7 to 18 years	P: General mental health assessment S: Depression (BDI-Y), anxiety (BAI-Y), anger (BANI-Y), disruptive behaviour (BDBI-Y), self-concept (BSCI-Y)	100 (5 inventories, 20 each)	5 - 10 minutes per inventory	Evidence of good concurrent validity for each of the individual inventories (except the anger inventory) with measures of those constructs (e.g., the depression inventory with the Children's Depression Inventory), and discriminative validity.	4-point Likert scale; 0, 1, 2, 3 (never, sometimes, often, always)
**2**	Brief Multidimensional Student's Life Satisfaction Scale	BMSLSS-PTPB-Y	Huebner, ES., Suldo, SM., Valois, RF., Drane, JW., & Zullig, K.	2003	To measure life satisfaction in children and adolescents.	8+ years	P: Family, school, friends, self, living environment	5	< 5 minutes	Evidence of construct validity with significant correlations as expected to measures of youth hope and youth symptom severity, and no relationship as expected to youth treatment outcome expectations.	7-point Likert scale; 1 - 7 (terrible to delighted)
**3**	Child Outcome Rating Scale, Young Child Outcome Rating Scale	CORS, YCORS	Duncan, BL., Brown, J., Sparks, JA., & Claud, DA.	2004	To measure of psychological distress in children and adolescents.	13+, 6 to 12 years	P: Psychological distress	4	< 5 minutes	Evidence of impressive internal consistency and test-retest reliability. The ORS and SRS show moderately strong concurrent validity with longer, more established measures of treatment outcome and therapeutic alliance. Feasibility of the ORS and SRS is high as they are ultra brief.	Child report: 10cm line with happy face at one end and sad face at the other, respondent asked to put mark on line. Session rating scale: 10cm line, respondent asked to put mark on line
**4**	Children's Quality of Life Questionnaire - Revised	KINDL-R	Ravens-Sieberer, U. & Bullinger, M.	1998	To assess quality of life in children and adolescents.	4 to 6, 7 to 13, 14 to 17 years	P: Quality of life	12, 24, 24	5 - 15 minutes	Evidence of reliability and validity. Reliability: internal consistency reliabilities (alphas) of 0.63-0.84. Validity: evidence of discriminative validity and extensive convergent validity.	4-6: 3-point scale (never, sometimes, very often) 7-13, 14-17: 5-point Likert scale (never, seldom, sometimes, often, all the time)
**5**	Children's Depression Inventory 2	CDI-2-Short, CDI-2-Long	Kovacs, M.	1992	To screen for depressive symptoms in children and adolescents.	7 to 17 years	P: Depression S (28-item): Emotional and functional problems, negative mood/physical problems, negative self-esteem, interpersonal problems, ineffectiveness	10, 28	5 - 10 minutes, 15 - 20 minutes	Evidence of well-established construct and discriminant validity. Internal consistency coefficients range from 0.71 to 0.89 and the test-retest coefficients range from 0.74 to 0.83.	3-point Likert scale; 0, 1, 2 (absence of symptoms, mild or probably symptoms, definite symptoms)
**6**	Children's Hope Scale	CHS, CHS-PTPB	Snyder, CR., Hoza, B., Pelham, WE., Rapoff, J., Ware, L., Danovsky, M., Highberger, L., Rubinstein, H., & Stahl, K.	1997	To assess dispositional hope in children and adolescents.	8 to 19 years	P: Cognition & development, hope S: Scale, agency, pathways	6,4	4 minutes	Evidence of reliability and validity. Evidence of acceptable test-retest reliability (Pearson correlation). Internal consistency acceptable (Chronbach's alpha).	6-point Likert scale; 1-6 (none of the time, a little of the time, some of the time, a lot of the time, most of the time, all of the time)
**7**	Columbia Impairment Scale	CIS	Bird, HR., Shaffer, D., Fisher, P., Gould, MS et al.	1993	To assess impairment and difficulties in performing usual activities due to due to a variety of health conditions and diseases, including mental or emotional problems.	9 to 17 years	P: Interpersonal relations, psychopathology, school performance, use of leisure time	13	5 minutes	Evidence of reliablity and validity. Excellent psychometric properties for target age population. High internal consistency scale (alpha=.78).	5-point Likert scale; 0,1,2,3,4,5 (no problem, some problem, <blank>, very bad problem, not applicable/don't know)
**8**	CRAFFT Screening Test (CAR, RELAX, ALONE, FORGET, FRIENDS, TROUBLE)	CRAFFT	Knight, JR. et al.	1999	To screen for high risk alcohol and other drug use disorders in adolescents.	<21 years	P: Substance abuse	6	1 - 2 minutes	Evidence of reliability and validity. Sensitivity: 76% to 92% Specificity: 76% to 94% PPV: 29% to 83% NPV: 91% to 98%.	Yes/no questions
**9**	Generalized Anxiety Disorder	GAD-2, GAD-7	Spitzer, RL; Kroenke, K; Williams, JB; Löwe, B.	2006	To screen and measure the severity of generalized anxiety disorder.	Unspecified	P: Generalized anxiety disorder S: Panic disorder, social anxiety disorder, post-traumatic stress disorder	2,7	< 5 minutes	Generalized Anxiety Disorder -Test Sensitivity: 89%, Test Specificity: 82% Panic Disorder - Test Sensitivity: 74%, Test Specificity: 81% Social Anxiety Disorder - Test Sensitivity: 72%, Test Specificity: 80% Post-Traumatic Stress Disorder (PTSD) - Test Sensitivity: 66%. Test Specificity: 81%.	4- point Likert scale; 0, 1, 2, 3 (never, sometimes, often, always)
**10**	Kutcher Adolescent Depression Scale	KADS-6, KADS-11	Kutcher, S.	2007	To assist in the public health and clinical identification of young people at risk for depression.	12 to 17 years	P: Depression S: suicidal ideation	6, 11	5 minutes	Evidence of good internal consistency (Cronbach's alpha) in both clinical (.74) and school (.80) samples.	4- point scale (hardly ever, much of the time, most of the time, all the time) and one question relating to the severity of suicidal ideation

**11**	Mood & Feelings Questionnaire	MFQ-Short, MFQ-Long	Angold, A. and Costello, EJ.	1987	To assess how the subject has been feeling or acting recently.	8 to 18 years	P: Depression	11, 32	5 - 10 minutes	Has received the Measurement Tools Rating of "A – Psychometrics Well-Demonstrated" based on the published, peer-reviewed research available.	3-point scale (most of the time/sometimes/not at all)
**12**	Patient Health Questionnaire - Youth	PHQ-2, PHQ-9	Spitzer, RL., Williams, JBW., Kroenke, K. et al.	1999	To detecting major depression in adolescents.	12 to 18 years	P: Depression	2, 9	5 minutes	Has been strongly supported for its applicability as a screening tool for adolescent depression in primary care as well as in pediatric hospital settings.	4-point scale (not at all, several days, more than half the days, nearly every day)
**13**	Pediatric Symptom Checklist	PSC-17	Bright Futures, Copyright © National Center for Education in Maternal and Child Health & Georgetown University	1988	To improve the recognition and treatment of psychosocial problems in children.	Parents of children and adolescents 4 to 16 years	P: Attention, externalizing and internalizing symptoms	17	5 - 10 minutes	Subscales have obtained reasonable agreement with validated and accepted parent-report instruments. Cronbach alpha was high for each subscale.	Parent report: 3-point scale (never, sometimes, often)
**14**	Personal Wellbeing Index - School Children	PWI-SC	Cummins, R., & Lau, A.	2005	To measure average well-being in school-aged children.	Unspecified age, children and adolescents	P: Quality of life, wellbeing S: Standard of living, health, achieving in life, relationships, safety, community-connectedness, future security, and spirituality/religion	8 +/- 1	5 minutes	A total of 22,912 Australian adults completed the measure between 2001 and 2004. Reliability: internal consistency reliabilities (alphas) ranging from .70 to .85. Test-retest (1-2 two week interval) reliability of .84. Validity: established construct validity and convergent validity correlation of r = .78 with the Satisfaction with Life scale. Evidence of validity with adolescents.	11-point Likert scale; 0-10 (very sad to very happy)
**15**	Reynolds Adolescent Depression Scale	RADS	Reynolds, WM.	1987	To screen adolescents in school or clinical settings for depression.	13 to 18 years	P: Depression (dysphonic mood, anhedonia/negative affect, negative self-evaluation, somatic complaints)	30	5 - 10 minutes	Reliability and validity studies included more than 9,000 adolescents and a clinical sample of adolescents with DSM-III-R™ or DSM-IV™ diagnoses who were evaluated in both school and clinical settings.	4-point Likert scale; 1,2,3,4 (almost never, hardly ever, sometimes, most of the time)
**16**	Strengths and Difficulties Questionnaire - Youth	SDQ-Y	Goodman, R.	1997	To assess the extent to which mental health problems have had an impact on aspects of the child's life.	3 to 17 years	P: Anxiety/mood (internalizing symptoms), externalizing symptoms, relationships & attachment, psychosocial functioning, cognition & development S: conduct symptom, emotional symptoms, hyperactivity, peer relationships, pro-social behaviour	25 (5 per domain)	5 - 10 minutes	Evidence of reliablity and validity in various populations and for a number of general mental health conditions. Sensitivity: 63% to 94%. Specificity: 88% to 98%.	3-point Likert scale; 0,1,2 (not true, somewhat true, certainly true)
**17**	Strong Souls	n/a	Thomas, A., Cairney, S., Gunthrope, W., Paradies, Y., Sayers, S.	2010	To assess social and emotional wellbeing in indigenous adolescents.	Unspecified age, aboriginal youth	P: Social and emotional wellbeing S: Anxiety, resilience, depression, suicide risk	25	10 minutes	Evidence of reliablity, validity, and cultural appropriateness as a tool for screening for SEWB among Indigenous young people in the Northern Territory. Selected as an appropriate tool to assess the social and emotional wellbeing (SEWB) of Indigenous adolescents participating in the longitudinal Aboriginal Birth Cohort (ABC) Study and was completed by 361 participants.	4-point scale (not much, sometimes, fair bit, lots of times)
**18**	The Children's Happiness Scale	n/a	Morgan, R (Children's Rights Director for England).	2014	To measure how happy a child or young person might be on a given day.	"Children in care, receiving social care support, and living away from home in boarding or other residential schools or colleges."	P: Subjective wellbeing, happiness	20	5 minutes	"We had the scale filled in by 2,186 children and young people...they were children in care, children getting support from children's social care services, children living away from home in all types of boarding schools and residential further education colleges, care leavers, or children living in residential special schools."	Tick the statement that applied to the child that day
**19**	Westerman Aboriginal Symptoms Checklist - Youth	WASC-Y	Westerman, T.	2003	To identify young Aboriginal people who are at risk of anxiety, depression and suicidal behaviours.	13 to 17 years, aboriginal youth	P: Depression, suicidal behaviour, substance abuse, impulsivity, anxiety, cultural resilience	53	> 20 minutes	Culturally and scientifically validated. Highly successful in identifying youth at risk of suicide.	n/a
**20**	Youth Self-Report	ABESA-YSR	Achenbach, TM.	1991	To assess problem behaviours along two "broadband scales": internalizing and externalizing. It also scores eight empirically based syndromes and DSM-oriented scales, and provides a summary of Total Problems. The measure assesses "Total Competency," which is a scale comprised of competency in activities, social functioning, and school performance.	11 to 18 years	P: General symptomatology, overall competence/functioning, mood and anxiety Symptoms S: Anxious/depressed, withdrawn/depressed, somatic complaints, social problems, thought problems, attention problems, rule-breaking behaviour, aggressive behaviour	112	> 15 minutes	Strongly supported content validity. Evidence that all items are significantly discriminant between demographically similar referred and non-referred youth. Validated in specific populations with complex trauma (5), physical abuse, sexual abuse, neglect, domestic violence, medical trauma, and other.	3-point Likert scale; 0,1,2 (not true, somewhat or sometimes true, very true or often true)
